# Fishing in the Cell Powerhouse: Zebrafish as A Tool for Exploration of Mitochondrial Defects Affecting the Nervous System

**DOI:** 10.3390/ijms20102409

**Published:** 2019-05-15

**Authors:** Gianluca Fichi, Valentina Naef, Amilcare Barca, Giovanna Longo, Baldassare Fronte, Tiziano Verri, Filippo M. Santorelli, Maria Marchese, Vittoria Petruzzella

**Affiliations:** 1 Molecular Medicine, IRCCS Stella Maris, Via dei Giacinti 2, 56028 Pisa, Italy; gianluca.fichi@gmail.com (G.F.); valentina.naef@gmail.com (V.N.); 2 Laboratory of General Physiology, Department of Biological and Environmental Sciences and Technologies, University of Salento, Via Provinciale Lecce-Monteroni, 73100 Lecce, Italy; amilcare.barca@unisalento.it (A.B.); tiziano.verri@unisalento.it (T.V.); 3 Department of Basic Medical Sciences, Neurosciences and Sense Organs, University of Bari ‘Aldo Moro’, Piazza Giulio Cesare 11, 70124 Bari, Italy; giovanna.longo@uniba.it; 4 Department of Veterinary Sciences, University of Pisa, viale delle Piagge 2, 56124 Pisa, Italy; baldassare.fronte@unipi.it

**Keywords:** zebrafish, mitochondria, nervous system development, neurodegenerative conditions

## Abstract

The zebrafish (*Danio rerio*) is a small vertebrate ideally suited to the modeling of human diseases. Large numbers of genetic alterations have now been modeled and could be used to study organ development by means of a genetic approach. To date, limited attention has been paid to the possible use of the zebrafish toolbox in studying human mitochondrial disorders affecting the nervous system. Here, we review the pertinent scientific literature discussing the use of zebrafish in modeling gene mutations involved in mitochondria-related neurological human diseases. A critical analysis of the literature suggests that the zebrafish not only lends itself to exploration of the pathological consequences of mitochondrial energy output on the nervous system but could also serve as an attractive platform for future drugs in an as yet untreatable category of human disorders.

## 1. Introduction

The term mitochondrial disease is classically used in reference to defects of the oxidative phosphorylation (OxPhos) system and defects of energy metabolism. Instead, the term mitochondrial medicine serves to group together, in a single category, the ample array of clinical presentations associated with these defects, which range from single organ failure to complex phenotypes, including neurodegenerative diseases and cancers [1]. The human mitochondrial proteome is believed to be made up of over 1500 proteins (Human MitoCarta (www.broadinstitute.org)) [2]. This reflects the intrinsic complexity of the organelle and makes it conceivable that dysfunctions of mitochondria give rise to highly heterogeneous clinical presentations. Patients may be affected at any age and with multisystem involvement, that often affects organs with high energy demands. However, how defects in mitochondria can cause such widespread range of human disorders remains to be unveiled. Mitochondria are dynamic and mobile organelles that play an essential role in several cellular processes, such as the generation and regulation of cellular energy, initiation of apoptosis, iron-sulfur cluster biogenesis, and calcium buffering [3,4]. The OxPhos is the process by which mitochondria produce the adenosine triphosphate (ATP) molecules needed by the cells. This system consists of five multimeric enzyme complexes (EC): Complex I (EC 1.6.5.3) or NADH:ubiquinone reductase, CI; Complex II (EC 1.3.5.1) or succinate dehydrogenase, CII; Complex III (EC 1.10.2.2) or quinol-cytochrome c (cyt c) reductase, CIII; Complex IV (EC 1.9.31) or cyt c oxidase (COX), CIV; Complex V (EC 3.6.14) or FoF1-ATPase, CV; and two electron transport carriers, namely, ubiquinone (coenzyme Q, CoQ) and cyt c [5]. The OxPhos is under a dual genetic control: thirteen of the key structural polypeptides that constitute the multimeric subunits of the respiratory chain complexes and ATP synthase are encoded by the mitochondrial DNA (mtDNA), in addition to two ribosomal RNAs (rRNAs) and 22 transfer RNAs (tRNAs), which are required to perform autochthonous protein synthesis [6]. Conversely, approximately eighty of the remaining proteins making up the OxPhos complexes are encoded by nuclear DNA (nDNA) genes. Although it encodes the basic machinery for protein synthesis, as well as replication, repair, and transcription, human mtDNA remains entirely dependent upon the nucleus for the provision of enzymes and accessory components. Hence, a genetic classification of OxPhos-related mitochondrial diseases distinguishes two broad categories: disorders due to mutations in the mtDNA, and obeying to the rules of mitochondrial genetics, and disorders due to mutations in the nDNA, which are governed by canonic Mendelian genetics [1]. Correct communication between the two genomes is crucial for mtDNA integrity, copy number regulation and mitochondrial protein production. Thus, not only nucleotide variants in the mtDNA itself, but also mutations in nuclear genes involved in mtDNA replication and maintenance might disrupt the integrity of the mitochondrial genome and influence its functional properties [7]. The pathological consequences impact mostly on tissues highly dependent upon ATP production such as heart, muscle, and particularly the central (CNS) and the peripheral nervous systems (PNS) [8].

Producing up to 90% of ATP molecules generated in the brain, the OxPhos is responsible for powering cell signaling and neuronal activity processes. It is not too surprising that mitochondria also play a key role in aging-related neurodegenerative disorders such as Parkinson’s disease (PD), Alzheimer’s disease, Huntington’s disease, and in amyotrophic lateral sclerosis [9,10]. Among the identified genes found to be associated with inherited forms of neurodegenerative disorders, at least half directly or secondarily affect one or several mitochondrial functions [11]. In cells, mitochondria actively fuse and divide (fission), branch, and change their size in a dynamic manner. This process, named mitochondrial network dynamics, seems indispensable to guarantee an appropriate population of healthy and functional mitochondria at every time [12]. Mitochondria network dynamics work in tight collaboration with the mitophagy pathway to ensure the quality control of these organelles [13]. Mitophagy is a selective form of macro-autophagy that degrades damaged or unnecessary mitochondria. Defects in this process have been reported as crucial at the onset and progression of neurodegenerative diseases where autophagic failure is one of the main factors contributing to neuronal cell death [14]. Neurons are particularly vulnerable to autophagic impairment as well as to mitochondrial dysfunction because of their high energy demands and to their post-mitotic non-proliferating nature [15]. Altogether, these considerations lead to the idea that mitochondria, as well as being a focus for a considerable amount of research, provide important clues to be potential target for therapies in a broad range of neurodegenerative diseases.

## 2. Zebrafish Resources to Mimic Human Diseases

The discovery of a large number of mitochondrial genes causing human diseases prompted the generation of a number of animal models, including simple invertebrate (*Saccharomyces cerevisiae*, *Caenorhabditis elegans* and *Drosophila melanogaster*) and more sophisticated mammalian models (such as *Mus musculus*), which replicate various aspects of neurological disorders caused by mitochondrial damage, and thus are considered useful to understand the pathophysiological mechanisms and to evaluate putative therapeutic candidates [16,17]. Nevertheless, there remains a need to achieve more efficient high-throughput screenings of vertebrate models in order to fully address the questions of mitochondrial physiology and function to study the effects of mitochondrial damage in individual organs and in the animal behaviors. Recently, the zebrafish has emerged as an excellent organism for studying a broad range of human genetic diseases, including those affecting the different functional structures of the CNS [18,19]. In this view, the zebrafish is attractive for several reasons including its particular biology and genetics. The zebrafish breed with high rates and big numbers of fertilized eggs, which are transparent, tiny, and mobile during the key developmental stages, can be easily obtained daily. The healthy zebrafish embryos develop rapidly and in synchrony, within one day of development, many key features of the CNS are detectable and can be studied up to the larval stage and beyond. Another major advantage of using zebrafish is the simplicity and effectiveness with which genes can be manipulated to allow biological observations in the cells of the developing embryos. Other advantages are the availability of zebrafish genome data [20] and the accessibility of over 80% of its gene structures in several publicly available databases (Ensembl (www.ensembl.org), Entrez (www.ncbi.nlm.nih.gov/gquery), UCSC Genome Browser (https://genome.ucsc.edu/), and ZFIN, the Zebrafish Model Organism Database (zfin.org)). Zebrafish genes show a high degree of synteny across vertebrate species as well as 50–80% homology with most human sequences. Comparison with the human reference genome sequences shows that approximately 70% of human genes have at least one obvious zebrafish ortholog [21]. In addition, molecular pathways are phylogenetically well conserved, and the basic physiology is similar to that of mammals [22]. Orthologs for most human genes can be easily identified by bioinformatics tools and manipulated to mimic human pathologies by gain- and loss-of-function approaches. However, it has to be noticed that zebrafish gene identification can be complicated by the fact that several genes are duplicated as a result of a whole genome duplication event that occurred during teleost fish evolution; this implies that in zebrafish specific functions could be partitioned between the two duplicated genes, or simply lost or disrupted for one of the genes, or even complemented if one of the two copies is disrupted [23]; nevertheless, in some cases duplication events enhance the possibility to obtain information on gain/loss of some gene functions.

A standard technique for studying embryo development is to knockdown a specific gene function by injecting gene-specific phosphorodiamidate morpholino oligonucleotides (MOs) in early embryonic stages [24]. MOs are chemically modified antisense oligonucleotides that can specifically bind messenger RNAs (mRNAs) and transiently block translation (when they are targeted to sequences near the initiation codon) or splicing (when they are targeted to exon-intron boundaries), thus providing gene knockdown in the zebrafish embryo. However, major limitations of this technique are the transient effects of MOs and the multiple off-target effects which fail to impact on the juvenile or adult phenotype [25]. More recently, the successful application of new technologies for genome editing, such as zinc finger nucleases [26,27], transcription activator-like effectors (TALENs) [28], and technologies based on clustered regularly interspaced short palindromic repeats (CRISPR) and CRISPR-associated protein 9 (Cas9) (CRISPR-Cas9) systems [29], has allowed more profitable targeted mutagenesis and functional gene ablation in zebrafish. Another approach is the undirected mutation-driven method using irradiation, retroviruses or the most commonly used chemical mutagens such as the DNA alkylating agent N-ethyl-N-nitrosourea (ENU) [30]. In the last years, the reverse genetics approaches, where DNA mutations can be studied without an observable phenotype, have been further improved by recent advances in genomic technologies and next-generation sequencing (NGS) [30,31]. The results of this progress have led to an increase in the number of novel zebrafish mutated lines.

## 3. Zebrafish Resources to Explore Mitochondrial Physiology

In the last decade, zebrafish modeling has been used to study various aspects of mitochondrial physiology, e.g., the dynamics of the cellular mitochondrial network, the mitochondrial life cycle, and mtDNA metabolism during development. The technologies applied to study these aspects in the zebrafish model have rapidly increased as shown in Table 1 and briefly described below.

Through a balance between fusion and fission, mitochondria maintain a web-shaped network in cells [32,33]. The study of mitochondrial morphology in the zebrafish was initially conducted using molecular probes [34] or immunofluorescent antibodies [35]. In order to study changes in mitochondrial morphology at the organism level in normal or disease conditions in real time, transgenic zebrafish model expressing mitochondrially-targeted fluorescent proteins (green/red) were created [36,37]. Several neurodegenerative diseases have been linked with deficits in mitochondrial axonal transport [38]. To study the “life cycle” of mitochondria and their dynamics in neurons, transgenic lines were generated using Gal4/UAS genetics that allows the expression of transgene labeled mitochondria [39] also in specific neural cells. By using this tool, studies on mitochondrial transport related to neurological disease were conducted in Rohon-Beard sensory neurons [40,41], retinal ganglion cells [42], motor neurons [43], and dopaminergic neurons [44] of zebrafish, developing different transgenic lines with fluorescently labeled mitochondria.

Recently, Mandal and colleagues (2018) have optimized a direct visualization of mitochondria and the analysis of their lifetime, health, and function in axons of the posterior lateral line (pLL) in zebrafish, using a panel of technologies that allows investigating in vivo several mitochondrial aspects in the zebrafish model (Table 1) [45].

Song and colleagues (2009) have investigated on mitochondrial reactive oxygen species (ROS) formation using the oxidative fluorescent dye dihydrorodamine-123 (DHR-123) [46], while the examination of mitochondrially derived ROS was conducted in zebrafish using a live cell redox sensor that identifies mitochondria-generated superoxides (MitoSOXTM) [47]. Biothilos, such as cysteine (Cys) and homocysteine (Hcy) have an important role in ROS homeostasis in mitochondria and are very sensitive to the oxidative stress [48]. The real-time monitoring of Cys and Hcy levels in zebrafish mitochondria allows monitoring their oxidative stress level. For this purpose, Yue et al. (2018) developed a ratiometric two-photon fluorescent probe (Mito-MQ) for measuring Cys/Hcy level in mitochondria and applied this technology in vivo, into 5-day-old zebrafish larvae [48]. All these studies provide a starting point for performing further mitochondrial studies under physiological or disease conditions in this relatively simple organism [49]. Micro-oxygraphy has been used in the whole organism, especially at the early stages of embryo development, to study bioenergetic metabolism and mitochondrial physiology related to neurodegenerative conditions [50]. Embryos have also been shown to lend themselves to the evaluation of the total number of mtDNA genomes at different developmental stages and to the assessment of the spatial expression of genes regulating mtDNA biogenesis and OxPhos complexes [51].

Due to the fact that many mitochondrial defects affect the nervous system, and many structure and function of the zebrafish CNS are very similar to the human ones [52], we recapitulated the scientific literature concerning the use of zebrafish for modelling neurological disorders caused by mitochondrial alterations. A critical review of what has been done to date might be of use for future investigations.

## 4. Modeling Mitochondrial Defects in Zebrafish

Mutations in mtDNA or nDNA genes that encode mitochondrial proteins cause a heterogeneous group of disorders primarily due to dysfunctions of the OxPhos and electron transport chain. However, other mutations that involve mitochondrial physiology, such as ion channels, quality control system, mitochondrial carriers, or cellular perturbations that affects mitochondria, such as mitochondrial transport cause an energy deficiency that affects high demand tissues including nervous system.

**Figure 1:**
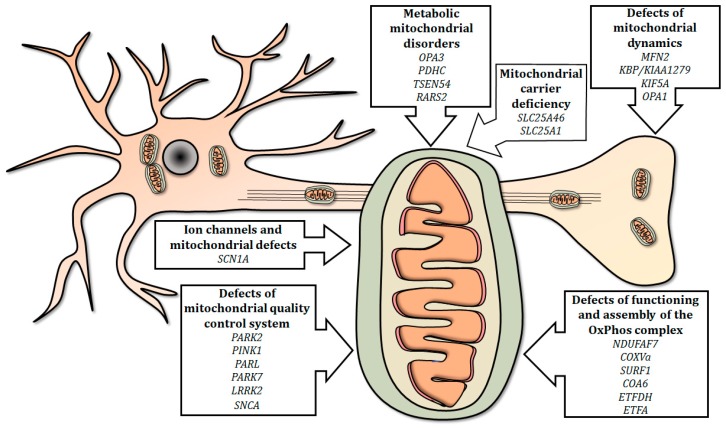
Summary of mitochondria-related genes involved in neurological human disorders, modeled in mutant zebrafish. Mutations in genes involved in mitochondrial electron transport chain, physiology, quality control, dynamics and metabolism, as well as mutations in selected genes encoding carriers and ion channels, can disrupt the integrity of the mitochondrion and/or influence its functions, thus determining the onset of neurological disorders.

In this review, we summarized the current use of the zebrafish as a relatively simple and efficient tool for studying the effects of gene mutations involved in mitochondria-related human neurological diseases (Figure 1).

Mutated genes modeled in zebrafish are presented in the following sections, covering six main topics; the list of genes investigated is summarized in Table 2.

### 4.1. Defects of Functioning and Assembly of the OxPhos Complex

The term “primary mitochondrial diseases” refers to a wide range of complex syndromes collectively characterized by impaired OxPhos function with clinical expression especially in the brain, muscle, and eyes [78,79]. The deficiency of OxPhos complex assembly factors causes several severe neurological disorders in human such as cardiomyoencephalopathy, leukoencephalopathy, psychomotor delay, seizures, and Leigh syndrome [5]. Currently, very few studies have been conducted on specific gene mutations to investigate these disorders using zebrafish models. Complex I is the largest enzyme of the mitochondrial OxPhos system and its deficiency caused by pathogenic mutations in genes encoding the structural subunits have been identified and associated with mitochondrial disorders [80].

A mutation on the methyltransferase NADH:Ubiquinone Oxidoreductase Complex Assembly Factor 7 (*NDUFAF7*), which is an assembly factor of complex I, has been found in association with the reduction of intracellular ROS and ATP levels and with the reduction of Complex I activity, and it has also been correlated with pathologic myopia [81]. Zurita Rendon and colleagues investigated the function of NDUFAF7 in models in vivo: *Ndufaf7* knockout mice and MO knockdown zebrafish. Delayed hatching times and morphological abnormality resulted by the disruption of the *NDUFAF7* paralogue gene, and the steady-state levels of complex I was specifically affected by the MO knockdown in zebrafish [54]. Furthermore, mutations of COX complex have been described in a number of human mitochondrial diseases with peripheral neuropathies. Among the mitochondrial diseases, COX deficiency can present with a number of different infantile clinical phenotypes including classical Leigh syndrome, fatal infantile COX deficiency, and hypertrophic cardiomyopathy and myopathy [82]. Most COX deficiencies in humans are related to defective function of structural or ancillary proteins making up the holocomplex, including the assembly genes *SCO2* and *SURF1*. Zebrafish morphants of the orthologs of either human *COXVa* or *SURF1* showed a profound histochemical defect of COX activity and impaired holoenzyme assembly [59]. As a consequence, morphants showed a dramatic increase in apoptosis in hindbrain and neural tube and exhibited a severe motility defect. By contrast, the heart of mutant zebrafish lacked apoptotic cells but showed increasingly poor performance over time, a phenotype consistent with tissue energy deficiency [55]. More recently, copper supplementation has been shown to rectify the disassembly pattern of the COX holocomplex in a zebrafish line where the COX assembly factor 6 (COA6) was knocked-down [56].

Multiple acyl-CoA dehydrogenase deficiency (MADD) is an autosomal recessive disorder, which is clinically heterogeneous; patients with this disease display multiple defects including neurological impairment. This condition is due to deficiency of any one of three proteins: the alpha (ETFA) and beta (ETFB) subunits of mitochondrial electron transfer flavoprotein, or the electron transfer flavoprotein dehydrogenase (ETFDH). The clinical pictures due to the different enzyme defects appear to be indistinguishable; each defect can lead to a range of mild or severe cases, depending presumably on the location and nature of the intragenic lesion [83]. Inactivation of the *etfdh* gene (*xavier* zebrafish mutant) resulted in severe metabolic abnormalities. In particular, there were biochemical abnormalities consistent with mitochondrial dysfunction, and increased neuronal proliferation caused by the activation of the PPARG-ERK pathway [46]. A new mutant strain termed *dark Xavier*, because of its enlarged, dark and fatty liver [57], was generated. This mutant strain, carrying mutations in *etfa*, displayed severe neurologic deficits including encephalopathy that is usually seen in patients with MADD, and recapitulated many key clinical and metabolic features seen in MADD patients such as multi-organ defects of the brain, liver and kidney.

### 4.2. Ion Channels and Mitochondrial Defects

Dravet syndrome (DS), a severe genetic form of epilepsy, has been associated with mutations in the sodium channel protein type 1 subunit alpha (SCN1A) [58]. Starting from the observation of mitochondrial defects in muscle biopsies in DS patients, OxPhos and mitochondrial glycolysis were studied in the *scn1Lab* zebrafish mutant, a zebrafish model of DS. Even though no defects of OxPhos complexes I–IV were observed in *scn1Lab* mutants, the authors noted a decreased expression of glycolysis related genes [58]. A decrease of complex I activity was suspected to be induced by the oxidative stress and post-translational oxidative modification caused by the spontaneous seizures, observed in these mutants [58].

### 4.3. Defects of Mitochondrial Quality Control System

The underlying causes of several neurological disorders converge on impaired mitochondrial physiology and maintenance. PD is a frequent neurological disorder caused by dopaminergic neuronal death in the *substantia nigra*, resulting in a reduced level of dopamine in the striatum that is the direct cause of motor dysfunction of PD patients [84]. PD-associated neuronal loss is strictly connected with localized mitochondrial dysfunction and oxidative stress before the clinical onset of motor symptoms [85]. Among gene products associated with hereditary PD we find those located within (PARKIN, PINK1, PARL, and DJ-1) or linked to (LRRK2) mitochondria. PARKIN and PINK1 are neuroprotective proteins which act together in a mitochondrial quality control pathway promoting the clearance of damaged mitochondria via autophagy [86].

PARKIN is an E3 ubiquitin ligase and like the human protein, zebrafish *parkin* is ubiquitously expressed throughout embryonic development and in adult tissues. PARKIN is involved in oxidative stress and the stable overexpression of *parkin* is able to protect fish against proteotoxic stress preventing cell death [67]. In zebrafish, loss of *parkin* elicits an approximately 20% loss of dopaminergic neurons in the ventral diencephalon. Morphants do not show any abnormal mitochondrial morphology, but mitochondrial complex I activity is spectrophotometrically reduced [59].

PINK1 is a ubiquitously expressed protein with an N-terminal mitochondrial-targeting motif and a conserved serine⁄threonine kinase domain, and two of its targets have been identified: TNF receptor-associated protein 1, which protects against oxidative stress [87], and DRP1, which promotes mitochondrial fission [88]. PINK1 seems to act similarly to PARKIN in oxidative stress conditions in particular, protecting neurons against stress-induced mitochondrial damage and apoptosis [89]. Zebrafish *pink1* is expressed ubiquitously in the brain, and its abrogation results in a selective decline of some important mRNAs, and only distinct groups of dopaminergic neurons are sensitive to loss of *pink1* in zebrafish [61]. The *pink1* null mutant zebrafish line, referred to as *pink^−/−^*, shows mitochondrial dysfunction and loss of dopaminergic neurons [62]. Differential transcriptomic investigations in *pink1* morphants [90] revealed global impairment of TGF-β signaling, retinoic acid receptor (RAR) activation, altered biogenesis of mitochondria, and, among the major hits, dysfunction of the hypoxia-induced signaling pathway when compared with a wild-type strain. Soman and co-workers have highlighted that the pharmacological and/or genetic inhibition of the inner mitochondrial membrane calcium uniporter is able to rescue loss of dopaminergic neurons in *pink^−/−^* mutants recovering the PD-phenotype [91]. Recently, this transgenic line (*pink^−/−^*) has been also exploited to develop new class of stress-dependent autophagy-stimulating drugs to prevent the loss of dopaminergic neurons in PD-zebrafish model [92].

*PARL* codes for an integral membrane protease of the inner mitochondrial membrane having a role in the normal trafficking and processing of PINK1 and PARKIN within mitochondria [93]. Moreover, *PARL* cleaves the optic atrophy 1 protein (OPA1) [94] that is a crucial protein involved in the fusion of inner mitochondrial membranes and in the formation of *cristae*, crucial for proper mitochondrial metabolism in early development [71]. Bioinformatics analyses revealed that in zebrafish there are two paralogues, *parla* and *parlb*, whose transcripts are both ubiquitously expressed during embryogenesis [63]. The PD-phenotype caused by the KD of both *parl* genes can be rescued by the overexpression of human *PARL* mRNA. Morphants showed that cell death likely occurred because *parl* is unable to prevent mitochondrial fission or to produce soluble OPA1, which prevents the widening of the cristae junction and inhibits the release of cyt c from the mitochondria [63].

*PARK7* encodes DJ-1 that is localized in the mitochondrial matrix; the zebrafish orthologue of DJ-1 is expressed in adult brain, muscle and gut, and is predicted to have high sequence homology of 83% with human DJ-1 protein [95]. Loss of human DJ-1 leads to an altered mitochondrial morphology contributing to the increased cellular sensitivity to oxidative stress [96]. In zebrafish, knockdown of *dj-1* does not cause decrease in number of dopaminergic neurons but leads to high susceptibility to programmed cell death activated by p53 [64]. Moreover, it is well known that the astrocytes, which outnumber neurons in the brain, have a key role in neuronal protection controlling redox homeostasis [84] producing neuron-protective factors [85]. The selective astroglial *dj-1* overexpression in transgenic zebrafish line Tg(gfap: egfp-2A-flag-zDJ-1) is able to protect fishes from neurological damage probably through the up-regulation of redox and inflammatory proteins regulated by Nrf2 activation [65].

LRRK2 has mainly cytoplasmic localization but associates also with the outer mitochondrial membrane. LRRK2 interacts with Parkin in cultured mammalian neuronal cells [97], and with PRDX3, an important antioxidant scavenger of hydrogen peroxide within mitochondria [98]. In human neuronal cell lines, the overexpression of LRRK2 causes mitochondrial fragmentation associated with increased levels of DLP1, a mitochondrial fission protein [99]. Knockdown of *lrrk2* in morphants zebrafish causes neurodegeneration and locomotion defects that can be rescued both by expressing the wild-type LRRK2 and with the administration of L-dopa, a drug commonly used for treating PD patients [66].

Another zebrafish model for PD has been created by overexpression of human α-synuclein (SNCA) in zebrafish peripheral sensory neurons that leads to a moderate cell death, whereas many axons exhibit diffuse or focal swellings [67]. Under basal conditions SNCA does not interact with mitochondrial membranes; nonetheless, ultrastructural studies reveal that a fraction of synuclein normally associates to mitochondria, and it can produce mitochondrial fragmentation and functionality impairment of the complex I activity [100]. The orthologue of human α-synuclein appears not to be present in the zebrafish genome [101]. However, the two synuclein isoforms, β and γ2-synuclein, can compensate the absence of α-synuclein, and their loss induces motor impairments and reduction of dopamine levels [68].

All of these observations lend further support to the notion that mitochondrial dysfunction plays a key role in zebrafish models of PD and that zebrafish represents a proficient model to investigate in vivo the alterations of mitochondrial physiology underlying neurological disorders.

### 4.4. Defects of Mitochondrial Dynamics

Mitochondrial dynamics, such as fission, fusion and transport, are fundamental processes for neurons because pre- and post-synaptic terminals need high amount of energy and Ca^2+^ buffering and are particularly susceptible to the deficiency of these systems [102]. When mtDNA and proteins accumulate defects, damaged mitochondria need to be replaced by newly ones assembled in the body of the cell and then transported along the axons. This anterograde and retrograde movement along the microtubules within neurons, vital to maintain energy homeostasis and essential neuronal activities, is mediated by motor/adaptor proteins and cytoskeletal elements [43,102]. Fission is necessary for the transport and to regulate mito-apoptosis of damaged mitochondria, while fusion allows the survival of damaged or senescent mitochondria by exchanging materials among mitochondria themselves [103].

Mutations in the *MFN2* (mitofusin 2) and *OPA1* genes, both involved in mitochondrial transport and/or fusion, cause two hereditary neurodegenerative disorders, respectively an axonal peripheral neuropathy (Charcot-Marie-Tooth Type 2, CMT2) and the dominant optic atrophy (DOA) [73]. *MFN2* codes for a dynamin-like GTPases located in the outer mitochondrial membrane, and its major function is promoting mitochondria movement and fusion. Vettori and colleagues (2011) developed a zebrafish model for the neuromuscular disorder CMT2 [69]. Patients with CMT2A are characterized by muscular atrophy, distal muscle weakness accompanied by distal sensory loss, foot deformities and gait impairment. Zebrafish knockdown of *mfn2* showed not dramatic alterations of the mitochondrial network structure but significant motor impairment or unresponsiveness to touch. Additional observations revealed a generalized impairment in the axonal structure of primary motor neurons, accompanied by the presence of shortened or missing axons, altered distribution of acetylcholine receptors with a reduction of the number and size of their clusters, which are important in neuromuscular junctions for the synaptic signals from the motor axon. Moreover, *mfn2* morphants displayed also an altered alignment of the myofibers [69]. Additionally, a spontaneous mutant strain harboring a nonsense mutation in mitofusin 2 (*mfn2*^L285X^) presented altered mitochondrial dynamics and a change of mitochondrial morphology [70]. The phenotype of this mutant strain revealed also a progressive motor dysfunction and alterations at the neuromuscular junction, associated with defective axonal transport of mitochondria, resembling the progressive motor dysfunction observed in CMT2 patients [70]. In order to study p.L76P and p.R94Q, two missense mutations in *MFN2*, mutant capped *MFN2* mRNAs were injected in the one-cell stage embryos of the new-created zebrafish line Tg(hb9: MTS-Kaede), characterized by the fluorescent labeling of motor neuron mitochondria [43]. Mutant capped *MFN2* mRNAs produced a significant reduction in the density of moving mitochondria for both mutations, but a significant reduction of mitochondrial density was observed in p.L76P overexpression in zebrafish motoneurons [43]. On the contrary, a reduction of the transport of mitochondria along the axon was observed in p.R94Q expressing larvae, suggesting a different mechanism of action of the two *MFN2* mutations [43].

*KIF5A* (Kinesin Family Member 5A) is another gene involved in mitochondrial transport, and mutations of this gene have also been associated with CMT2 [38]. KIF5A belongs to the Kinesin superfamily proteins (KIFs), which are microtubule-based molecular motors. Mammals possess three KIF5s: Kif5B that is ubiquitously expressed, and KIF5A and KIF5C that are neuron-specific [104]. In zebrafish there are five *kif5* genes (*kif5Aa*, *kif5Ab*, *kif5Ba*, *kif5Bb*, and *kif5C*) and *kif5Aa*, *kif5Ab*, and *kif5C* are the most expressed in neural tissues [104]. Campbell and colleagues observed that only the *kif5Aa* mutated zebrafish line showed larval lethality and sensorimotor deficits similar to CMTs [38].

Kinesin proteins have an important role during neuronal development, but additional factors affect their regulation [37]. Kif1-binding protein (KBP/KIAA1279) binds to the motor domain of the KIF1B and KIF1C, and homozygous mutations of KBP cause Goldberg–Shprintzen syndrome, a severe disorder characterized by neurological symptoms, mental retardation, and disruption of white matter tracts. Lyons and colleagues (2008) investigated the *KPB* paralogue gene in zebrafish *kbp*^st23^ mutants and demonstrated its key role in the regulation of axonal cytoskeleton organization, regulation of axonal outgrowth speed, localization of axonal cargo and maintaining of axonal longer-term integrity. The consequence on mitochondria of the KBP mutation in zebrafish was a mislocalization and a reduction in the number of axonal mitochondria [37].

*OPA1*, a dynamin-related GTPase, is involved in the fusion of the outer and inner mitochondrial membranes, and gene mutations are associated with the autosomal dominant optic atrophy (ADOA) [71]. ADOA is characterized by progressive loss of visual acuity, desensitization of central visual field, optic nerve pallor, and eventual blindness [71]. In zebrafish, the *opa1* morphants morphologically showed a reduction of the eyes size. In morphant 72 hpf (hours-post-fertilization) embryos a reduction of the startle response and of the locomotor activity were also observed associated to bioenergetics defects [71]. Furthermore, morphants showed upregulation of *pgc1a*, the orthologue of the master regulator of mitochondrial biogenesis, at 24 and 48 hpf, suggesting increased mitochondrial biogenesis [71]. The *opa1* morphants allowed elucidating the connection between *opa1* depletion, mitochondrial dysfunction, and development [71].

These results implicate that modeling in zebrafish proteins regulating mitochondrial dynamics might serve to study the distribution of mitochondria in early stages of development and to improve knowledge on the role of mitochondrial networks in neurodegeneration.

### 4.5. Mitochondrial Carrier Deficiency

Mitochondrial carriers (MCS) are transport proteins that allow small molecules to shuttle between mitochondria and cytoplasm playing a central role in mitochondrial function to regulating cellular metabolism [105] (and literature cited therein). Few studies have addressed the role of mitochondrial carriers in neurological disorders and no less the functions of many human carriers still remain better to be characterized. In mammals, MCS comprise the transporters of the 53-member canonical *SLC25* family and a lesser number of identified non-canonical transporters.

SLC25A1, the first member of the solute carrier family SLC25, is the mitochondrial citrate carrier that mediates the exchange of mitochondrial citrate/isocitrate with cytosolic malate [106,107]. SLC25A1 is involved in fatty acid and sterol biosynthesis, gluconeogenesis and glycolysis [106], maintenance of chromosome integrity and regulation of autophagy [108,109]. The identification of patients harboring mutations in SLC25A1 and presenting congenital myasthenic syndrome 23 (CMS23) [72] or the more severe combined D-2- and L-2-hydroxyglutaric aciduria (D2L2AD) [110,111] phenotype confirms the relevance of this protein in humans. SLC25A1 dysfunction interferes with brain, eye, and possibly neuromuscular development, in addition to causing a distinctive urinary organic acid profile. Notably, knocking down the two zebrafish *SLC25A1* orthologues (i.e., *slc25a1a* and *slc25a1b*) by injection of antisense MOs in fertilized eggs mirrors human CMS23 in terms of variable brain, eye, and cardiac involvement, and leads to abnormalities in the neuromuscular junction, regardless of the severity of the phenotype [72]. MO-injected embryos display altered tail morphology, and impaired swimming and touch-evoked escape responses at 48 hpf. Histologically, muscle morphology is normal but neuromuscular junction development is abnormal. Motor axon terminals show short and erratic outgrowth toward the muscle fiber with no evidence of complete synapse formation, suggesting presynaptic nerve terminal abnormalities. Knockdown embryos often show edema of the hindbrain, heart, yolk sac and tail. Abnormal heart development is observed with increased severity of phenotype with reduced blood flow to the tail [72].

SLC25A46, another member of the mitochondrial solute carrier family SLC25, was found to correspond to UGO1, a mitochondrial fusion factor in *Saccharomyces cerevisiae*. Previously, genome studies had linked SLC25A46 to atopic dermatitis but a high expression of the *SLC25A46* transcript had been observed in the spinal cord, cerebellum, and optic chiasm neurons of patients [73]. Severe *slc25a46* zebrafish morphants showed a curly-tail morphology while wild-appearing larvae display an altered swimming [73] associated to significantly shorter axon tracts in motor neurons and fewer neuronal processes in spinal cord neuropil of larvae. The dendritic degeneration observed in *slc25a46* morphants was consistent with CMT2 pathology. In degenerating neurons, an incomplete mitochondrial fission was observed, and mitochondria resulted aggregated and predominantly in the apical portion of the soma [73]. Interestingly, different mutations in *SLC25A46*, that cause loss of function of the mutant protein, were also found in patients with the pontocerebellar hypoplasia (PCH) [74]. SCL25A46 seems to act as a pro-fission component regulating mitochondrial dynamics in humans and zebrafish.

### 4.6. Metabolic Mitochondrial Disorders

Metabolic disorders causing neurological impairment have successfully been modelled in zebrafish. To date, these disorders include Costeff syndrome, Barth syndrome, pyruvate dehydrogenase complex (PDHC) deficiency, and PCH.

Costeff syndrome is a neuro-ophthalmological disorder characterized by increased urinary excretion of 3-methylglutaconic acid and 3-methylglutaric acid, early-onset optic atrophy, and later occurrence of spasticity and extra-pyramidal features [112]. Costeff syndrome is caused by mutations in *OPA3* that encodes an inner mitochondrial membrane protein. In zebrafish *opa3* mRNA is expressed in the optic nerve and retinal layers, brain, inner ear, heart, liver, intestine, and swim bladder. Morphants do not show vision loss, hyperreflexia or spasticity characteristic of the human disorder but a penetrant behavior resembling the ataxia and extrapyramidal (bradykinesia) features seen in Costeff syndrome [75].

The PDHC is a nuclear-encoded mitochondrial matrix multienzyme complex that catalyzes the irreversible conversion of pyruvate into the acetyl form of coenzyme A (acetyl-CoA), thus playing a central role in linking glycolysis to the tricarboxylic acid cycle and lipogenic pathways [113]. Deficient biochemical activity causes fatal neonatal conditions including congenital lactic acidosis, growth retardation and Leigh syndrome. A spontaneous zebrafish mutant of PDHC deficiency, exhibits phenotypes similar to human patients and the administration of a ketogenic diet resembling the leading therapy proposed for children with PDHC deficiency is able to recover the normal condition [76].

Currently, PCH classification comprises 11 types based on distinct genetic causes and clinical features. The disease is associated with prenatal onset, hypoplasia/atrophy of the cerebellum, hypoplasia of the ventral pons, progressive microcephaly and variable neocortical atrophy [114]. Zebrafish models of PCH have been generated through the knockdown of *tsen54* and *rars2*, two genes encoding proteins associated with tRNA splicing and tRNA aminoacylation. Studies in morphants suggest a common disease pathway may exist between *TSEN54*- and *RARS2*-related PCH, which may involve a tRNA processing-related mechanism [77].

All these studies reveal that the zebrafish modeling is sensitive to metabolic insults from embryonic stages, and thus it may be useful in order to understand disease mechanisms and critical to develop therapeutic options in inborn errors of metabolism affecting mitochondrial functions [22].

## 5. Zebrafish as In Vivo Model to Test New Compounds in Mitochondrial Related Neurological Disorders

The zebrafish represents a valuable model for investigating the molecular mechanisms underlying the neurotoxicity of agents affecting the normal neuronal mitochondrial physiology and for testing potential neuroprotective compounds. As mentioned above, defects in mitochondrial biogenesis, mitochondrial dynamics, and mitochondrial respiratory chain activities lead to a several human diseases; below, we provide several new models for mitochondrial dysfunction which could have great potential for diagnostics and therapies in neuronal mitochondrial-related diseases.

The impairment of the OxPhos system affects directly mitochondrial function causing several disease phenotypes. The inhibition of ATP production can be induced in zebrafish by exposure to 2-4 dinitrophenol (DNP), a weight loss agent with significant toxicity, used in research as an uncoupler of OxPhos [115]. Past mid-embryogenesis embryos treated by DNP show a strong decrease in ATP levels and developmental defects. The neural tissues emerge particularly affected by DNP, which inhibits motor neuron axon arbor outgrowth and proper formation of the retina [116].

COX, the terminal oxidase enzyme of the mitochondrial respiratory chain, needs copper (Cu) as an essential micronutrient required for its assembly and activity. Mutations in genes related to the delivery for Cu to COX leads to a respiratory deficiency and fatal mitochondrial disease. In a zebrafish model of Cu deficiency, low nanomolar quantity of the anticancer drug elesclomol (ES) is able to recover phenotypes associated with Cu deficiency by increasing cellular and mitochondrial Cu content [117].

Pyruvate oxidation defects are also among the causes of mitochondrial diseases mainly due to deficiency of subunits of PDHC. In zebrafish, *noa* mutants (carrying a mutation in the pdh-e2 subunit) treated with 4-phenylbutyrate (PBA), a histone deacetylase inhibitor usually employed in urea cycle disorders, showed a significant decrease in melanophore expansion, more active locomotion, and lower levels of lactate and pyruvate than untreated mutants. These findings prompted evaluation of PBA in human mutant cells and in a mouse model of PDHC deficiency [118]. Another zebrafish mutant (*noir* strain, harboring a stop codon mutation in the *pdh* subunit E1 β gene) showed a retinal phenotype and reduced locomotion and fatigue, and the phenotype improved upon treatment with a ketogenic diet causing an increase in acetyl-CoA production, able to bypass the metabolic block [119].

Recently, the idebenone metabolite 6-(9-carboxynonyl)-2,3-dimethoxy-5-methyl-1,4-benzoquinone (QS10) was used to recover of respiration and to allow zebrafish wild-type embryos survival after the treatment with the neurotoxin Rotenone, an inhibitor of complex I [120]. Currently, idebenone was proposed as a first-line therapy in Leber’s hereditary optic neuropathy (LHON) syndrome because its capacity to improve visual defects in a subset of patients [121,122].

It was widely described [123] that also in zebrafish some structural analogs of dopamine, such as 6-hydroxydopamine (6-OHDA) and 1-methyl-4-phenyl-1,2,3,6-tetrahydropyridine (MPTP), selectively affect dopaminergic neurons inducing a PD-like phenotype. In particular, the metabolite of MPTP, 1-methyl-4-phenylpyridinium (MPP+), inhibits complex I and impairs mitochondrial respiration [124]. These findings allowed generating numerous zebrafish PD-models to test new potential compounds with neuroprotective activity.

The transgenic zebrafish line Tg(ETvmat2: GFP) showed a significant neurodegeneration of dopaminergic neurons and locomotor dysfunction upon treatment with MPTP [125]. This model has widely been used as an in vivo tool for screening neuroprotective agents for PD, such as Paenolum, that is the main component derived from the bark of the *Paeonia suffruticosa*, acting as a strong antioxidant and reducing the mitochondrial cell death pathway [126].

Moreover, it has been shown that the exposure of 24 hpf zebrafish embryos with melatonin added together with MPTP or added once MPTP removal, prevents and recovers the parkinsonian phenotype restoring mitochondrial normalcy and the brain function in zebrafish. Melatonin is able to restore in a dose-specific manner the gene expression and the normal function of the PD-related genes *park2/pink1/park7* loop, and also the normal motor activity of the embryos [127]. Furthermore, sleep disorders constitute major nonmotor features of PD and several trials indicate that melatonin and its analogs are useful in treating disturbed sleep in PD patients [128,129].

Zhang and co-workers (2017) developed a multifactorial zebrafish drug-screening platform discovering a series of piperazine phenothiazines, with neuroprotective activity that rescued stress-induced mitochondrial dysfunction thus preventing dopaminergic neuron loss in a zebrafish model of pink1 deficiency [92]. In particular, trifluoperazine (TFP) is able to increase autophagic flux acting downstream of, or parallel to, pink1/parkin to restore TFEB translocation, which is a master regulator of the autophagy-lysosomal pathway [92].

Another zebrafish model of PD was generated by treating the fish with 6-OHDA, and the marine-derived compound 11-dehydrosinulariolide (11-de) increases cytosolic or mitochondrial *dj*-1 expression, and then activates the downstream Akt/PI3K, p-CREB, and Nrf2/HO-1 pathways [130]. Several marine-derived natural compounds have been approved for clinical use [131]. Huang and co-workers summarized those effective for the treatment of PD and that have been approved in clinical trials [132]. All these evidences suggest that zebrafish can be a powerful instrument for a full understanding of mitochondrial pathogenesis related to the nervous system.

## 6. Materials and Methods

A literature search was performed on original articles published by the 28th November 2018, which were identified from PubMed using the terms <<zebrafish>> AND <<neurological dysfunction [All Fields] >> and <<zebrafish [All Fields]>> AND <<mitochondrion [All Fields]>>as queries. These searches yielded respectively 80 and 503 matches in PubMed. We also performed an active search of the references listed in publications discussing neurological diseases associated with mitochondrial damage and the use of zebrafish. Altogether, we critically re-evaluated 571 articles net of repetitions but 526 were excluded because they did not present direct research on zebrafish, mitochondria and neurological disorders. Overall, 47 articles were included in the review.

## 7. Conclusions

Mitochondria are dynamic organelles that carry out many cell functions and, in particular, they are known as the “powerhouse” of the cell, generating ATP via OxPhos. The high number/density of mitochondria within neurons to attend their high energy demands provides a rationale for the sensitivity of the CNS to energy deficits due to mitochondrial impairment.

The large number of novel genotypes and the increased number of mutants developed in the last years, as well as the new tools developed for analyzing mitochondrial lifetime, health, and function in zebrafish combined with the use of MO and CRISPR-Cas9 systems, have allowed to investigate and understand the role of several genes involved in human mitochondrial-related neurological disorders.

The list of identified mutations associated with mitochondrial alterations in neurological disorders is still growing thanks to the use of NGS in the diagnostics of neurological human diseases. Zebrafish has proven itself to be an applicable and versatile model to investigate mutations responsible of mitochondrial disorders.

In parallel, one of the key characteristics of the zebrafish is that it can serve as a platform in high-throughput screenings performed to assess drug efficacy or to analyze off-target effects in a whole-organism context. Many drugs for treating human diseases have comparable effects in zebrafish embryos, and it is likely that additional compounds identified by large-molecule screening in zebrafish will be worth testing in mammalian systems. The zebrafish can be exploited as a practical tool for performing more detailed and rapid studies to shed light on the effects and consequences (inert, ameliorating, or detrimental) during development, which have rarely been analyzed in proper clinical trials. In addition, the model can serve while assessing safety and efficacy of drugs in their early stages of development.

## Figures and Tables

**Table 1:** Tools used for the investigation of mitochondrial physiology.

Tools	Investigation	Refs
MitoTracker Deep Red and MitoTracker Green FM	Mitochondrial activity in skin	[34]
mitochondrial localization sequence- enhanced GFP embryos	Mitochondrial morphology in real time	[53]
Mito:mCherry	KBP/KIAA1279 function	[37]
Oxidative fluorescent dye dihydrorodamine-123 (DHR-123)	Mitochondrial ROS formation	[46]
Seahorse XF24 Extracellular Flux Analyzer	Mitochondrial bioenergetics	[50]
Relative quantification by RT-PCR and immuno-probes	Genes involved in mtDNA replication and transcription and genes of the OxPhos system	[51]
MitoSOX^TM^	Examination of mitochondria-derived ROS	[47]
MitoFish, Tg(elavl3.2:Gal4-VP16)mde4/Tg(UAS-E1b:mYFP,mitoCFP)mde	Time-lapse imaging of mitochondrial transport in Rohon-Beard sensory neurons	[41]
MitoDsRed transgenic line: reporter UAS–mitoDsRed–polyA crossed to isl1(ss):Gal4VP16:14XUAS-GFP fish.	Morphology and motility of mitochondria in somatosensory neurons	[40]
MitoGFP	In vivo study of mitochondria in retinal ganglion cell of *kif5Aa* mutant	[42]
Tg(hb9:MTS-Kaede)	Mitochondrial dynamics in motor neurons in CMT2A mutants	[43]
Tg(otpb:Gal4); Tg(UAS:mtPAGFP:mtDsRed2)	Measurement of mitochondrial transport in dopaminergic neurons	[44]
Anti-mitochondrial membrane 20 (TOM20) and anti-mitochondrial aspartate aminotransferase (mAAT)antibodies	Study of ES1, a mitochondria-enlarging factor in cones	[35]
5kbneurod:mito-TagRFP plasmid	Role for Actr10 in dynactin-mitochondria interaction	[36]
Tg(5kbneurod:mitomEos)y586	Mitochondrial lifetime and the timeline of mitochondrial turnover and temporal dynamics of mitochondrial gain and loss from axon terminals	[45]
5kbneurod:mito-Timer	ROS production, an indicator of mitochondrial health	[45]
Vital dye TMRE	Measurement of potential/pH across the mitochondrial inner membrane	[45]
G-GECO1; Tg(5kbneurod:GGECO)nl19; R-GECO1; Tg(5kbneurod:mito-R-GECO)nl20	Mitochondrial calcium buffering in neurons	[45]
ATP:ADP dual ratiometric sensor PercevalHR	Productivity of mitochondria in the cell body and axon terminal of pLL sensory axons	[45]
Mito-MQ	Oxidative stress by Real-time monitoring Cys and Hcy levels	[48]

**Table 2:** Human genes related to mitochondrial disorders affecting the nervous system modeled in zebrafish.

Gene	Function	Modeling Zebrafish	Phenotype	Refs
Defects of functioning and assembly of OxPhos complex				
*NDUFAF7*	Assembly factor of Complex I	MO-mediated knockdown	Delayed hatching times and morphological abnormality	[54]
*COXVa*	Structural component of COX	MO-mediated knockdown	Developmental defects in endodermal tissue, cardiac function and swimming behavior	[55]
*SURF1*	Assembly factor of COX	MO-mediated knockdown	Developmental defects in endodermal tissue, cardiac function and swimming behavior	[55]
*COA6*	Cu-delivery pathway for COX assembly	MO-mediated knockdown	Heart developmental defects	[56]
*ETFDH*	Electron transfer flavoprotein dehydrogenase	*xavier* mutant zebrafish line	Altered energy metabolism, dysregulated ROS production, increased aerobic glycolysis, motility defects, abnormal glial patterning, reduced motor axon branching and neuromuscular synapse number	[46]
		MO-mediated knockdown	Bent tail and reduced heartbeat, aberrant swimming behavior, and reduced neuromuscular synaptogenesis	[46]
*ETFA*	Receiving of electrons from dehydrogenases involved in fatty acid β-oxidation, amino acid and choline metabolism	*dark xavier (dxa^vu463^)* mutant zebrafish line	Increased number of neural progenitor cells and accumulation of neutral lipid and cerebroside sulphate in brain, hepatic steatosis and dysmorphic kidneys, and hypomyelination	[57]
Ion channels and mitochondrial defects				
*SCN1A*	Brain-specific voltage-activated sodium channel	*scn1Lab* mutant zebrafish line	Increased behavioral seizure activity and increased glycolytic rate	[58]
Defects of mitochondrial quality control system				
*PARK2*	E3 ubiquitin ligase	MO-mediated knockdown	High susceptibility to the MPP^+^, Dopaminergic loss neurons and complex I deficiency	[59]
		Overexpression of parkin in transgenic zebrafish cell lines	Protective reaction against cell death induced by proteotoxic stress	[60]
*PINK1*	Mitochondrial serine/threonine kinase	MO-mediated knockdown	High sensitivity to MPTP, increase of ROS level, activation of apoptotic signaling	[61]
		*pink1* null mutant zebrafish line	PD-phenotype and altered biogenesis of mitochondria	[62]
*PARL*	Presenilin-associated rhomboid-like protein that is part of the PINK1 and Parkin pathway. In zebrafish are present two paralogs (*parla* and *parlb*)	MO-mediated knockdown	High rate of mortality in early larvae, mis-patterned dopaminergic neurons in morphants	[63]
*PARK7*	Pleiotropic function: transcriptional regulator, antioxidant scavenger, redox sensor and roles as a chaperone with protease activity	MO-mediated knockdown	Neurons are highly sensitive to hydrogen peroxidase	[64]
		Transgenic line Tg(gfap:egfp-2A-flag-zDJ-1)	Astrocytic *dj-1* overexpression protects mutants from neurological damage induced by the PD-related neurotoxin MPP^+^	[65]
*LRRK2*	Cytosolic protein	MO-mediated knockdown	Neurodegeneration and locomotion defects	[66]
*SNCA*	Soluble protein primarily expressed in the neural tissue. In zebrafish are present only two synuclein isoforms, β and γ2-synuclein	Overexpression of human α-synuclein	Changes in mitochondrial density and morphology, mitochondrial fragmentation, reduced mitochondrial motility and ensuing synaptic dysfunction and degeneration	[67]
		Double MO-mediated knockdown	Motor impairments and reduction of dopamine level	[68]
Defects of mitochondrial dynamics				
*MFN2*	Promotion of mitochondria movement and fusion	MO-mediated knockdown	Reduction of the survival rate, motor impairment or unresponsiveness to touch. Generalized impairment in the axonal structure of primary motor neurons. Presence of shortened or missing axons, altered distribution of acetylcholine receptors. Altered alignment of the myofibers	[69]
		*mfn2^L285X^* mutant zebrafish line	Altered swimming and progressive loss of motor function. Alterations at the neuromuscular junction. Altered mitochondrial dynamics and change in mitochondrial morphology	[70]
		Overexpression of *MFN2* carrying the c.281G>A (R94Q) and c.227T>C (L76P) mutations in transgenic zebrafish cell lines	Reduction of mitochondria transport along the axon in p.R94Q expressing larvae. Reduction of density of moving mitochondria in the case of p.L76P overexpression.	[43]
*OPA1*	Fusion of the outer and inner mitochondrial membranes	MO-mediated knockdown	Developmental delay, decreasing of the blood circulation velocity, and reduction of the eye size and the heart rate. Reduction of the startle response and bioenergetics defects	[71]
*KBP/KIAA1279*	Binding protein of the kinesin motor proteins KIF1B and KIF1C	*kbpst^23^* mutants	Delay in development of peripheral axons. Axons degeneration. Reduction in myelination. Disorganization of the axonal cytoskeleton. Reduction in the number of axonal mitochondria.	[37]
		MO-mediated knockdown	Axonal defects in peripheral and central nervous systems	[37]
*KIF5A*	Transport processes	*kif5Aa* mutant zebrafish line	Hyperexcitability, peripheral polyneuropathy, and axonal degeneration	[38]
Mitochondrial carrier deficiency				
*SLC25A1*	Mitochondrial solute carrier family	MO-mediated knockdown	Altered tail morphology, impaired swimming and touch-evoked escape responses. Abnormal neuromuscular junction development. Short and erratic outgrowth toward the muscle fiber of the motor axon terminals with no complete synapse formation. Hindbrain, heart, yolk sac, and tail edema. Abnormal heart development with reduced blood flow to the tail (in severe phenotypes)	[72]
*SLC25A46*	Mitochondrial solute carrier family	MO-mediated knockdown	Curly-tail morphology and altered swimming. Shorter axon tracts in motor neurons and fewer neuronal processes in spinal cord. Degenerate neurons with incomplete mitochondrial fission, mitochondria aggregated and misplaced.	[73]
		MO-mediated knockdown	Poor motility and the loss of spinal motor neurons.	[74]
Metabolic mitochondrial disorders				
*OPA3*	Inner mitochondrial membrane protein	MO-mediated Knockdown	Increased 3-methylglutaconic acid (MGC), and features mimicking movement disorders	[75]
*PDHC*	Nuclear-encoded mitochondrial matrix multienzyme complex	Spontaneous zebrafish mutant (*noa strain*, carrying a mutation in the pdh-e2 subunit)	Retinal abnormalities, defects of synaptic transmission and of light adaptation in cone photoreceptor cells, premature death, lethargy, expanded melanophores, and absence of feeding behavior	[76]
*TSEN54*	Subunit of the tRNA-splicing endonuclease (TSEN) complex	MO-mediated Knockdown, mutagenesis strategy	Brain hypoplasia and loss of structural integrity, increased cell death, and early lethality in zebrafish	[77]
*RARS2*	Mitochondrial arginyl-tRNA synthetase gene	MO-mediated knockdown	Brain hypoplasia, cell death and neurodegeneration	[77]

## Abbreviations

6-OHDA	6-hydroxydopamine
acetyl-CoA	Acetyl form of coenzyme A
ADOA	Autosomal dominant optic atrophy
Cas9	CRISPR-associated protein 9
CMT2	Charcot-Marie-Tooth Type 2
CNS	Central nervous system
COA	COX assembly factor
CoQ	Coenzyme Q
COX	Cytochrome c oxidase
CRISPR	Clustered regularly interspaced short palindromic repeats
Cys	Cysteine
cyt *c*	cytochrome *c*
DNP	2-4 dinitrophenol
DOA	Dominant optic atrophy
DS	Dravet syndrome
EC	Enzyme complexes
ENU	N-ethyl-N-nitrosourea
Hcy	Homocysteine
MADD	Multiple acyl-CoA dehydrogenase deficiency
MCS	Mitochondrial carriers
MO	Morpholino oligonucleotide
MPP^+^	1-methyl-4-phenylpyridinium
MPTP	1-methylmethyl-4-phenylphenyl-1,2,3,6-tetrahydropyridinetetrahydropyridine
mRNA	Messenger RNA
mtDNA	Mitochondrial DNA
nDNA	Nuclear DNA
NGS	Next-generation sequencing
OxPhos	Oxidative phosphorylation
PBA	4-phenylbutyrate
PCH	Pontocerebellar hypoplasia
PD	Parkinson’s disease
PDHC	Pyruvate dehydrogenase complex
pLL	Posterior lateral line
PNS	Peripheral nervous system
ROS	Reactive oxygen species
rRNA	Ribosomal RNA
TALEN	Transcription activator-like effector
tRNA	Transfer RNA
